# Overexpression of TGF-ß1 in Macrophages Reduces and Stabilizes Atherosclerotic Plaques in ApoE-Deficient Mice

**DOI:** 10.1371/journal.pone.0040990

**Published:** 2012-07-19

**Authors:** Kurt Reifenberg, Fei Cheng, Carolin Orning, Jeanine Crain, Ines Küpper, Elena Wiese, Martina Protschka, Manfred Blessing, Karl J. Lackner, Michael Torzewski

**Affiliations:** 1 Central Laboratory Animal Facility, University Medical Center, Johannes Gutenberg-University, Mainz, Germany; 2 Institute of Clinical Chemistry and Laboratory Medicine, University Medical Center, Johannes Gutenberg-University, Mainz, Germany; 3 Center for Biotechnology and Biomedicine, Veterinary Faculty, University of Leipzig, Leipzig, Germany; 4 Department of Laboratory Medicine, Robert-Bosch-Hospital, Stuttgart, Germany; Washington University School of Medicine, United States of America

## Abstract

Although macrophages represent the hallmark of both human and murine atherosclerotic lesions and have been shown to express TGF-ß1 (transforming growth factor β1) and its receptors, it has so far not been experimentally addressed whether the pleiotropic cytokine TGF-ß1 may influence atherogenesis by a macrophage specific mechanism. We developed transgenic mice with macrophage specific TGF-ß1 overexpression, crossed the transgenics to the atherosclerotic ApoE (apolipoprotein E) knock-out strain and quantitatively analyzed both atherosclerotic lesion development and composition of the resulting double mutants. Compared with control ApoE^−/−^ mice, animals with macrophage specific TGF-ß1 overexpression developed significantly less atherosclerosis after 24 weeks on the WTD (Western type diet) as indicated by aortic plaque area *en face* (p<0.05). Reduced atherosclerotic lesion development was associated with significantly less macrophages (p<0.05 after both 8 and 24 weeks on the WTD), significantly more smooth muscle cells (SMCs; p<0.01 after 24 weeks on the WTD), significantly more collagen (p<0.01 and p<0.05 after 16 and 24 weeks on the WTD, respectively) without significant differences of inner aortic arch intima thickness or the number of total macrophages in the mice pointing to a plaque stabilizing effect of macrophage-specific TGF-ß1 overexpression. Our data shows that macrophage specific TGF-ß1 overexpression reduces and stabilizes atherosclerotic plaques in ApoE-deficient mice.

## Introduction

TGF-β (transforming growth factor-β) family members TGF-β1, TGF-β2, and TGF-β3 are widely expressed cytokines with pleiotropic functions which operate by binding to two types of cell surface receptors (types II and III). TGF-β1 is known for its important role in development, proliferation, migration, differentiation, and extracellular matrix biology, but also for its potent immunomodulatory effects [1], [2]. TGF-β1 is synthesized by several cardiovascular cell types involved in atherogenesis including endothelial cells, monocytes/macrophages and T cells [3]; and may exert anti-atherogenic actions [4]. This view has received support by clinical studies indicating a negative correlation between plasma TGF-β1 concentrations and the extent of atherosclerotic lesions [4], [5]. Experimental studies, in which TGF-ß1 effects have been inhibited in blood vessels of atherosclerosis-prone ApoE (apolipoprotein E) knock-out mice by application of specific antibodies [6] or of recombinant soluble type II receptor [7] resulted in exacerbation of atherosclerosis. Accordingly, the reverse experiment by tetracycline-dependently overexpressing TGF-ß1 in the heart and plasma of atherosclerotic mice [8] found a reduction of atherosclerosis and could thus support the atheroprotective properties of TGF-ß1. In an attempt to specifically address the influence of TGF-ß on atherogenesis, Goyova et al. [9] and Robertson and co-workers [10] used crossing experiments or bone marrow transplantation to introduce transgenic T-cells into atherosclerosis-prone mice in which the TGF-ß1 signal transduction was specifically inhibited by overexpression of a dominant negative form of the type II receptor. Although these two studies found opposite effects on lesion size, they were in general agreement that blockade of TGF-β signalling in T cells increased vascular inflammation (which itself is certainly atherogenic). However, the experimental approach of T-cell specific inhibition of TGF-ß1 signalling by overexpression of a dominant negative type II receptor must generally be considered as limited since the immunological phenotype of such mouse strains differs depending on expression pattern and promoters used [9], [10] and is significantly more moderate compared to mice where the TGFßR2 gene was T-cell specifically targeted using the conditional Cre/LoxP technology [11].

Although macrophages represent the hallmark of atherosclerotic lesions and although macrophages secrete TGF-ß1 [12], and are involved into the activation of TGF-ß1 [13] and express TGF-ß receptors [14], so far it has not been experimentally addressed whether the pleiotropic cytokine TGF-ß1 may influence atherogenesis by a macrophage specific mechanism. To fill this gap, we developed transgenic mice with macrophage specific TGF-ß1 overexpression, crossed the transgenics to the atherosclerotic ApoE knock-out strain and quantitatively analyzed the atherosclerotic lesions of the resulting double mutants.

## Materials and Methods

### Generation and Characteristics of Transgenic Mice

For generation of transgenic mice with macrophage-specific TGF-ß1 overexpression the cDNA of the simian (*Cercopithecus aethiops*) TGF-ß1 gene [15] was cloned downstream of the human SRA (scavenger receptor A) promoter/enhancer (generously provided by Christopher K. Glass, University of California, San Diego, CA) [16], [17]. The resulting fusion transgene was referred to as SRA-TGF-ß1. The SRA-TGF-ß1 transgene construct was microinjected into C57BL/6 (B6) embryos according to standard protocols and the resulting founders expanded by crossing to the B6 strain. SRA-TGF-ß1 transgenic mice could be discriminated from non-transgenic littermates by PCR amplification of transgene specific sequences using primers 5′-TAC CTA CCA GTT CTG CGC CT-3′ and 5′-CTG TTG GCG AAG ACA CTC CT-3′, respectively.

For expression analyses of SRA-TGF-ß1 mice lungs were quick-frozen in liquid nitrogen and stored at −80°C until RNA extraction. The tissue was ground in a liquid nitrogen-cooled mortar with pestle. Total RNA was isolated using Tri-Reagent (Sigma-Aldrich, Taufkirchen, Germany). The concentration of the RNA was determined by spectrophotometry (NanoDrop, peQLab, Erlangen). DNA was digested with 1–2 Units DNAse (Roche)/µg RNA for 30 min at room temperature. RNA was precipitated with 1/10 volume of 3 M sodium acetate, pH 5.5 and two volumes of 100% ethanol. Reverse transcription was performed using ‘Revert Aid H Minus First Strand cDNA Synthesis Kit’ (MBI-Fermentas, St. Leon-Rot, Germany). Real-time PCR with SYBR green detection was performed using iQ™ SYBR® Green Supermix (Bio-Rad, Munich, Germany) and a SmartCyclerII-System with fluorescence detection (Cypheid, Sunnyvale, USA). The following primers were used for PCR analysis: spT4∶5′-TAC CTA CCA GTT CTG CGC CT-3′ and GH2∶5′-CTG TTG GCG AAG ACA CTC CT-3′ (GenBank Accession No. J03071; position 6162-6143) for TGF-ß expression analysis; HPRT-for: 5′-GTTGGATACAG GCCAGACTTTGTTG-3′ and HPRT-rev: 5′-GATTCAACTTGCGCTCATCTTAGGC-3′ (GenBank Accession No. NM_013556; positions 660-684 and 822-798) for the expression analysis of hypoxanthine-phosphoribosyltransferase (HPRT). HPRT was used as housekeeping gene for the normalization of the expression data. The relative quantification of the transcripts was done by the 2^(−ΔΔCt)^ method.

### Dietary Amplification of Atherosclerosis

Beginning at an age of 8 weeks female transgenic SRA-TGF-ß1 ApoE^−/−^ experimental mice as well as ApoE^−/−^ controls were administered an atherogenic WTD (Western type diet, Ssniff Spezialdiäten GmbH, Soest, Germany) for 8, 16 or 24 weeks, respectively. The WTD diet contained 21% (wt/wt) fat and 0.15% (wt/wt) cholesterol.

All laboratory mice were maintained at the Central Laboratory Animal Facility under strict SPF (specific pathogen free) conditions. Animals were housed in accordance with standard animal care requirements and maintained on a 12/12 hour light-dark cycle. Water and food were given *ad libitum*. Genetic authenticity of all strains was monitored commercially (KBioscience, Hoddesdon, UK) using the SNP (single nucleotide polymorphism)-based marker set previously developed by The Jackson Laboratory [18].

All animal work performed in this study was conducted according to the national guidelines and was reviewed and confirmed by an institutional review board/ethics committee headed by the local animal welfare officer (Prof. Kempski) of the University Medical Center (Mainz, Germany). The animal experiments were finally approved by the responsible national authority, which is the National Investigation Office Rheinland-Pfalz (Koblenz, Germany). The Approval ID assigned by this authority is AZ 23 177-07/G 07-1-003.

### TGF-β1 Protein, Lipoprotein and Lipid Analyses

Serum TGF-β1 protein was measured with both a human and mouse TGF-β1 ELISA kit (R&D Systems, Cat. Number DY240 and DY1679). Murine sera were diluted 1∶3 before quantitative cholesterol and triglyceride analyses. Quantitative cholesterol determinations were conducted using a colorimetric assay (CHOD-PAP, Roche Diagnostics, Mannheim, Germany). Triglycerides were determined by quantifying free glycerine originating from hydrolytic cleavage (GPO-PAP, Roche Diagnostics).

### Tissue Preparation and Quantitative Morphometry of Atherosclerotic Lesion Development

Mice were euthanized by exposure to carbon dioxide. Peritoneal cavities were opened and the cadavers fixed in 4% buffered formaldehyde. Hearts and aortas were resected *en bloc* down to the iliac bifurcation and carefully cleaned of perivascular adipose tissue under a dissection microscope (Leica MZ6; Leica, Bensheim, Germany). The aortic arch and the rest of the aorta from below the arch down to the iliac bifurcation were separated. Longitudinal sections of the aortic arch were stained with trichrome and computer-assisted (Image Pro Discovery; Media Cybernetics, Silver Spring, MD) measurement of plaque size was performed as described [19]. The maximal lesion area and thickness of the inner aortic arch intima (lesser curvature) of each mouse were used to compute averages per group. The rest of the aortae from below the arch down to the iliac bifurcation was opened longitudinally and stained with freshly prepared Sudan IV. Sudan stained atherosclerotic lesions *en face* were then quantified using Photoshop-based image analysis (Version 8.0.1, Adobe Systems Inc., San Jose, CA) as described [20].

### Immunohistochemical and Histochemical Analyses

Immunostaining of murine tissues with the rat or murine MAbs was performed using the VECTASTAIN Elite ABC Kit or Vector M.O.M. immunodetection kit (Vector Laboratories, Burlingham, CA). Serial 5 µm-thick sections of the paraffin-embedded aortic arch, lung, liver or spleen were deparaffinized in xylene. All slides were treated with 3% H_2_O_2_ to block endogenous peroxidase activity. Slides were incubated consecutively with 5% normal serum to block non-specific binding, primary antibody rat anti-mouse F4/80 (clone CI:A3-1 (F4/80), Acris Antibodies, 1∶100), murine anti-smooth muscle α-actin (clone 1A4, Sigma, 1∶100) or polyclonal goat anti-TGFβ1 (sc-146G, Santa Cruz Biotechnologies, 1∶500) for 1 hour, biotin-conjugated secondary anti-mouse antibody for 30 minutes and avidin-biotin-peroxidase reagent for 45 minutes at room temperature. The reaction products were identified by immersing the slides in DAB (diaminobenzidine tetrachloride) to give a brown reaction product. The slides were then counterstained with hematoxylin and mounted. Negative controls included replacement of the primary antibody by an irrelevant isotype-matched antibody (DAK-GO1, DakoCytomation). Collagen content was analyzed by picrosirius red and polarized light microscopic imaging. Percent-positive area for immunohistochemical or picrosirius red staining was quantified by Photoshop-based image analysis as described [21], [22], [23]. Briefly, pixels with similar chromogen characteristics were selected with the “magic wand” tool and the “select similar” command, and the ratio of the positively stained area to the total lesion area studied was calculated with the “histogram” command in Photoshop. Splenic macrophages were quantified as the average number of F4/80 positive cells per field (100× magnification) from at least 6 randomly selected fields per section. All quantitative morphometric and immunohistochemical data were collected independently by two experienced operators blinded to the mice genotypes.

### Statistical Analyses

Data were analyzed with SPSS 17.0 for Windows. Since most of the outcome parameters determined in this study did not follow a normal distribution as judged by the Shapiro-Wilk test statistical analyses were performed with the non-parametric Mann-Whitney U test. Serum concentrations of lipid contents are presented as mean (± standard deviation). Data concerning lesion composition (macrophages, SMCs, collagen) are presented as box plots with median, interquartile range, minimum and maximum. Differences were considered as significant when the p-value fell below a limit of 0.05.

## Results

### Generation and Expression Analysis of Transgenic Mice with Macrophage-specific TGF-ß1 Overexpression

**Figure 1:**
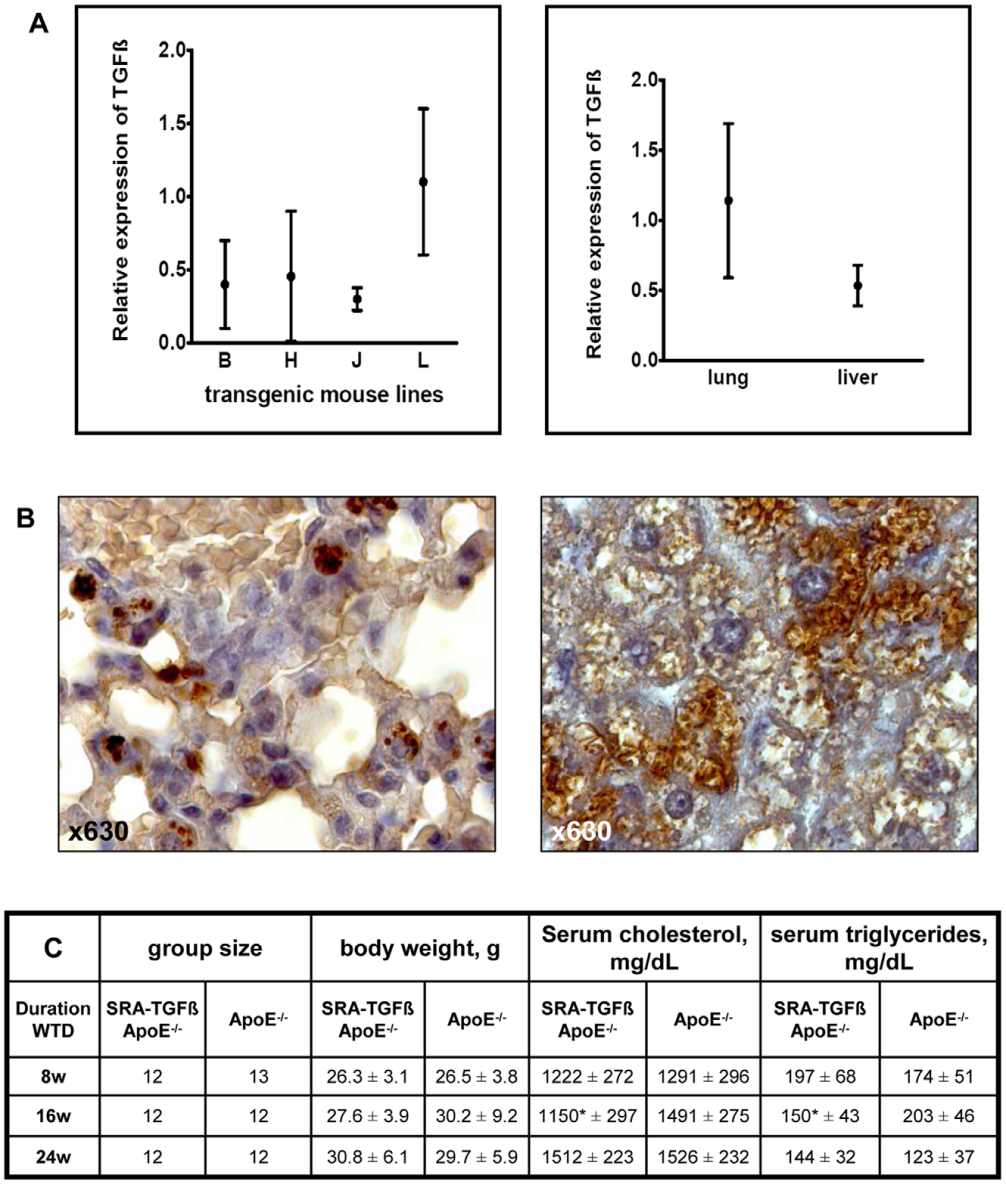
A Generation and expression analysis of transgenic mice with macrophage-specific TGF-ß1 overexpression. Comparative quantitative RT-PCR analyses was performed with total lung RNA isolated using Tri-Reagent (Sigma-Aldrich, Taufkirchen, Germany) of at least three 8 weeks old transgenic mice of strains SRA-TGF-ß1-B (B), SRA-TGF-ß1-J (J), SRA-TGF-ß1-L (L) and SRA-TGF-ß1-H (H) (left panel). Strain SRA-TGF-ß1-L (L) was used for additional comparative quantitative RT-PCR analysis of total liver RNA (right panel). In both panels, HPRT was used as housekeeping gene for the normalization of the expression data. The relative quantification of the transcripts was done by the 2^(-ΔΔCt)^ method. **B** Demonstration of TGF-ß1 expression by immunohistochemistry in both lung (left panel) and liver tissue (right panel) by using a polyclonal goat anti-TGFß1 antibody. **C** Group size, body weights, serum cholesterol and serum triglyceride concentrations of SRA-TGF-ß1 ApoE^−/−^ and ApoE^−/−^ mice. Data are presented as means ± standard deviation.

Twelve transgenic founder mice could be obtained by microinjection of the SRA-TGF-β1 construct. The founders were propagated to four transgenic strains referred to as SRA-TGF-β1-B, SRA-TGF-β1-J, SRA-TGF-β1-L and SRA-TGF-β1-H. Comparative quantitative RT-PCR analyses performed with total lung RNA of at least three 8 weeks old transgenic mice per strain showed that lineage SRA-TGF-β1-L had the highest TGF-β1 expression characteristics (Fig. 1 A, left panel) and we thus selected this strain for all further experiments. As the simian (*Cercopithecus aethiops*) TGF-β1 gene product could not be detected reliably by the available ELISA kits, we further documented TGF-β1 expression in the tissue by additional comparative quantitative RT-PCR analysis with total liver RNA (Fig. 1 A, right panel). Finally, TGF-β1 expression was corroborated by immunohistochemistry in both lung and liver tissue (Fig. 1 B). To clarify whether TGF-β1 overexpression affects the number of total macrophages in the mice we assessed splenic macrophage number in both SRA-TGF-ß1-L ApoE^−/−^ mice and ApoE^−/−^ controls. However, there were no statistical significant differences between the double mutants and the apoE knockout controls (118.4±60.43 versus 139.9±70.9 macrophages per HPF, p = 0.276).

### Serum Lipids and Lipoproteins and Atherosclerosis Lesion Progression

Female SRA-TGF-ß1-L ApoE^−/−^ experimental mice (also referred to as SRA-TGF-ß1 ApoE^−/−^ animals, see above) and ApoE^−/−^ controls were placed on a WTD beginning at an age of 8 weeks for 8, 16, and 24 weeks, respectively. As depicted in Fig. 1 C the mean body weights, serum cholesterol and serum triglyceride concentrations of SRA-TGF-ß1 ApoE^−/−^ and ApoE^−/−^ mice did not differ significantly with the exception of the cholesterol and triglyceride levels of mice having received WTD for 16 weeks.

**Figure 2:**
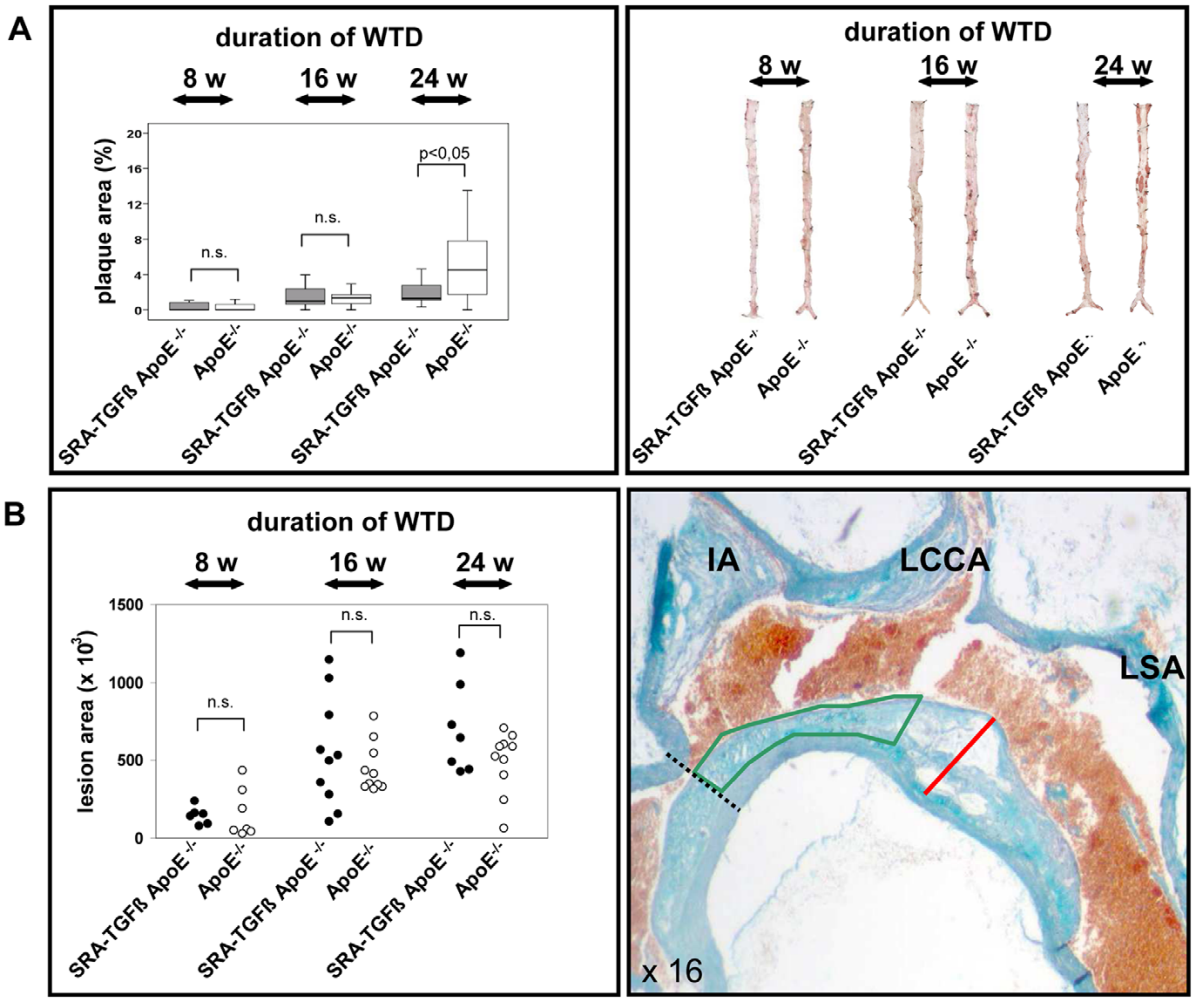
Quantification of atherogenesis *en face* (A) and in the aortic arch (B) in the mouse model of TGF-ß1 overexpressing macrophages after 8 weeks (8 w), 16 weeks (16 w) and 24 weeks (24 w) on the WTD, respectively. **A, left panel** Box and whisker diagrams (median, interquartile range, minimum, and maximum; n.s.  =  not significant) of aortic plaque area (%) of SRA-TGF-ß1 ApoE^−/−^ (grey) and ApoE^−/−^ mice (white). **A, right panel** Representative Sudan-stained aortas *en face* of SRA-TGF-ß1 ApoE^−/−^ and ApoE^−/−^ mice. **B, right panel** Hearts of SRA-TGF-ß1 ApoE^−/−^ and and ApoE^−/−^ mice were resected, and measurement of plaque size in longitudinal sections of the aortic arch stained with trichrome was performed as follows: a 2-mm segment of the lesser curvature of the aortic arch was defined proximally by a perpendicular axis dropped from the right side of the innominate artery origin (dashed line) and the aortic-arch wall area subtended by this 2-mm stretch of intima (green line) was calculated for each section of all mice by computerized image analysis. In addition, the aortic-arch intima thickness (red line) was determined on this same segment of the lesser curvature [1]. IA, innominate artery; LCCA, left common carotid artery; LSA, left subclavian artery. Note, that no statistically significant differences of lesion area could be detected between SRA-TGF-ß1 ApoE^−/−^ (black circles) and ApoE^−/−^ (white circles) mice (non-parametric Mann-Whitney U test, n.s.  =  not significant, **B, left panel).**

After 8 and 16 weeks on the WTD, respectively, no significant differences of mean atherosclerosis *en face* could be detected between SRA-TGF-ß1 ApoE^−/−^ animals and ApoE^−/−^ controls. Compared with control ApoE^−/−^ mice, however, transgenic mice developed significantly less atherosclerosis after 24 weeks on the WTD, as indicated by plaque area *en face* of the aorta from below the arch down to the iliac bifurcation (Fig. 2 A). Quantification of the thickness of the inner aortic arch intima (lesser curvature) revealed no significant differences in control mice compared with transgenic mice after 8, 16 and 24 weeks on the WTD (8 weeks: 118.4 µm ±60.43 µm versus 139.9 µm ±70.9 µm, p = 0.276; 16 weeks: 222.2 µm ±62.1 µm versus 218.6 µm ±45.7 µm, p = 0.883; 24 weeks: 263.9 µm ±38.2 µm versus 291.4 µm ±59.9 µm, p = 0.548).

### Phenotype Analysis of Atherosclerotic Lesions

**Figure 3:**
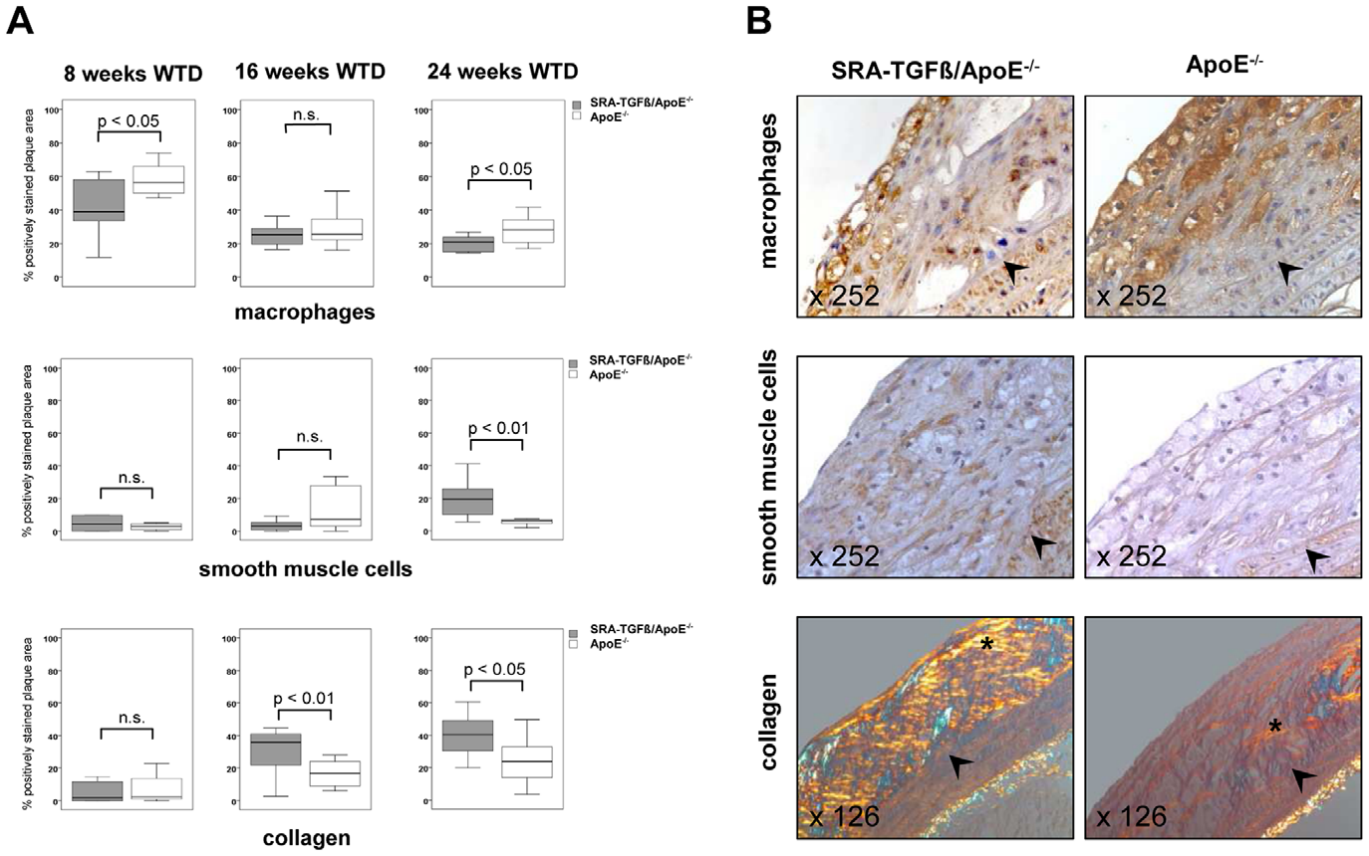
Phenotypic analysis of atherosclerotic lesions in SRA-TGF-ß1 ApoE^−/−^ mice. **A** Box and whisker diagrams (median, interquartile range, minimum, and maximum; n.s.  =  not significant) of the quantification of macrophages (upper panel), SMCs (middle panel) and collagen (lower panel) in atherosclerotic lesions of SRA-TGF-ß1 ApoE^−/−^ females and isogenic ApoE^−/−^ controls after 8, 16 and 24 weeks on the WTD, respectively. **B** Representative histological slides of atherosclerotic lesions located in the inner aortic arch intima (lesser curvature) of SRA-TGF-ß1 ApoE^−/−^ mutants and ApoE^−/−^ controls after 24 weeks on the WTD. The slides have been stained for macrophages, SMCs, and collagen by using a rat anti-mouse F4/80 antibody (upper panel), a mouse anti-smooth muscle α-actin antibody (middle panel), and picrosirius red with subsequent polarization (lower panel). Percent-positive area for macrophages (upper panels, asterisks), SMCs (middle panels, brown-stained areas), and collagen (lower panels, areas with yellow, green, orange, or red polarized colour) were quantified by Photoshop-based image analysis. The aortic lumen is to the upper left corner. The demarcation between intima and media is indicated by black arrowheads. In the lower panel, note that the adventitial tissue (asterisk) also polarizes after picrosirius red staining (internal positive control).

Quantification of the maximal area of the inner aortic arch intima (lesser curvature) revealed no significant differences in control mice compared to double-mutant mice after 8, 16 and 24 weeks on the WTD, respectively (Fig. 2 B). However, complementary to the quantitative atherosclerosis *en face* results in particular after 24 weeks on the WTD, the SRA-TGF-ß1 ApoE^−/−^ double-mutant mice with macrophage-specific TGF-ß1 overexpression showed multiple significant differences in terms of plaque composition compared to ApoE^−/−^ controls. Thus, the lesions of SRA-TGF-ß1 ApoE^−/−^ mice included significantly less macrophages at 8 and 24 weeks on the WTD (Fig. 3 A and 3 B, upper panels), significantly more SMCs at 24 weeks on the WTD (Fig. 3 A and 3 B, middle panels) and significantly more collagen at 16 and 24 weeks on the WTD (Fig. 3 A and 3 B, lower panels) compared to the respective ApoE^−/−^ controls pointing to a plaque stabilizing effect of macrophage-specific TGF-ß1 overexpression.

**Figure 4:**
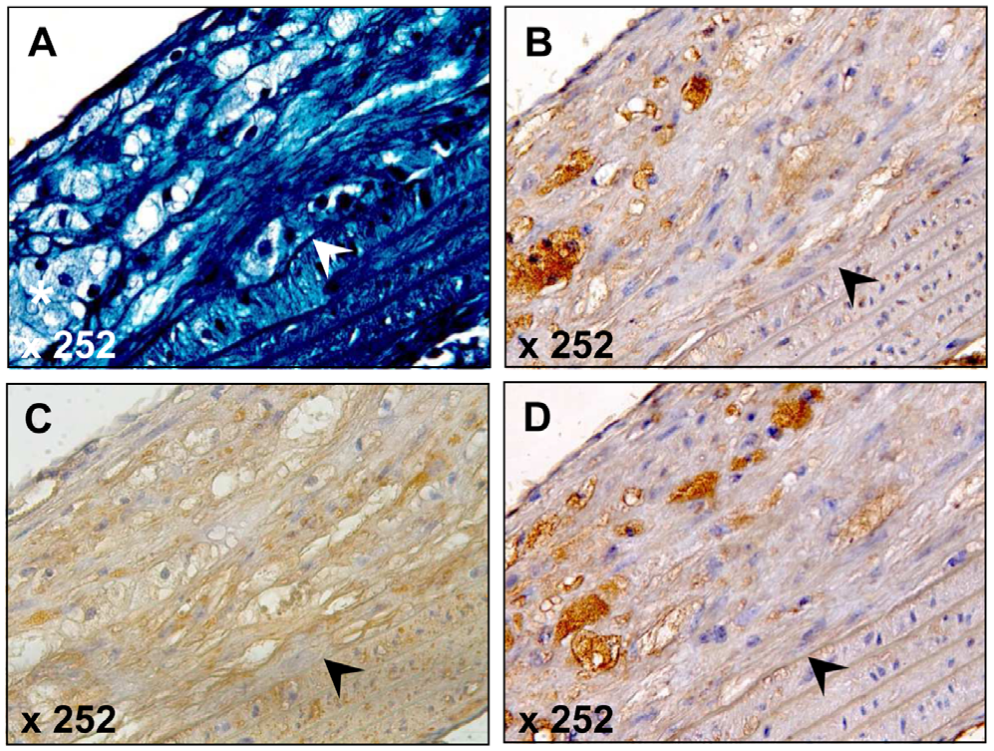
Representative examples of TGFß1 expression in atherosclerotic lesions of SRA-TGF-ß1 ApoE^−/−^ mice after 24 weeks on the WTD: Atherosclerotic lesions of the inner aortic arch intima (lesser curvature) were stained with trichrome (upper left panel), rat anti-mouse F4/80 (upper right) for macrophages, mouse anti-smooth muscle α-actin (1A4) (lower left panel) and goat anti-human TGFß1 (lower right panel). The lumen is to the upper left corner. The demarcation between intima and media is indicated by an arrowhead.

To assess the spatial distribution of TGF-ß1 expression in the murine atherosclerotic lesions, we performed specific TGF-ß1 staining with a polyclonal antibody together with specific macrophage and SMC staining of serial sections. As shown in Fig. 4, there was a close association of TGF-ß1 and macrophage staining presenting evidence that TGF-ß1 expression is restricted to macrophages and that the macrophages make TGF-ß1 protein.

## Discussion

Strong clinical evidence [4], [5] and animal experimental confirmation [6], [7], [8] have been provided for an anti-atherogenic effect of the pleiotropic cytokine TGF-ß on atherogenesis whereas the underlying mechanism remains to be elucidated.

In a first attempt to clarify this important question the groups of Goyova and co-workers [9] and Robertson et al. [10] have investigated whether the athero-protective effects of TGF-ß may depend on intact TGF-ß signalling in T-cells. This experimental approach is well-founded since on the one hand T-cells have been detected in atherosclerotic plaques albeit at low quantities [24] and on the other hand T cell function has been found to be strongly regulated by TGF-ß [25]. Interestingly, the most prominent effect of T cell specific abrogation of TGF-ß signalling in T cells of atherosclerotic mice as found by Robertson and co-workers [10] was a significant acceleration of atherosclerosis, whereas the group of Goyova [9] observed a decrease of lesion size in combination with a plaque phenotype more vulnerable to rupture. At a first glance, these results are conflicting. But although these two studies found opposite effects on lesion size, they were in general agreement that blockade of TGF-ß signalling in T cells increases vascular inflammation (which itself is certainly atherogenic). However, we must be aware that the immunological phenotype of transgenic mice with T-cell specific overexpression of a dominant negative type II TGF-ß receptor differs in dependence of the transcriptional control elements used and is strongly different to that of mice with a T cell specific TGFßR2 gene defect [11]. In this context it has to be emphasized that it is still unclear whether adaptive immunity plays an important role in atherogenesis at all [26], [27]. Thus, experimental studies performed in atherosclerosis-prone immunodeficient mice supported the view of a pro-atherogenic function of the adaptive immune system only in some cases [28], [29], [30] but found only minor or no effects in others [26], [27], [31], [32]. Further, no specific epitopes leading to T cell activation during atherogenesis have been identified [33].

In addition to T cells, TGF-ß and its receptors are abundantly expressed by macrophages and SMCs in human atherosclerotic lesions during development of fatty streaks and subsequent atheroma [34]. Notably, macrophages principally have been shown to express TGF-ß receptors [14] and TGF-ß is considered as a potent macrophage modulating agent [35]. Finally, monocytes may play a larger role than T and B cells during atherogenesis in the ApoE-deficient mouse [32].

Given the above-mentioned controversial views about the contribution of the immune system to atherogenesis and T cell specific abrogation of TGF-ß signalling in murine atherosclerosis; and since macrophages represent the hallmark of both human and murine atherosclerotic lesions, the present study was aimed to specify the role of TGF-ß in atherogenesis by transgenetical overexpressing of an active form of the cytokine in atherosclerotic lesions of ApoE knockout mice using a strong macrophage-specific promoter.

After 24 weeks on an atherogenic WTD, the TGF-ß1 overexpression approach led to both smaller atherosclerotic lesions (Fig. 2) and a phenotype characterized by an increase of collagen deposition and SMC infiltration as well as a decrease of macrophage content (Fig. 3), which was not due to a decrease of the number of total macrophages in the mice as demonstrated by quantification of splenic macrophages.

Because there is no map of mice atherosclerotic lesion appearance and accumulation, nor “standard curves” for lesion quantitation, laboratories may establish their own protocol for induction of atherosclerosis, based on gender, diet, and age [36]. However, according to a plethora of studies in apoE-deficient mice, a time course up to 24 weeks ensures that we were able to assay the development of lesions at all stages, from the foam cell lesion to the fibrous plaque. In particular, the WTD guarantees the development of mature atherosclerotic plaques much like the plaques that occur in humans in a reasonably short time [37], [38]. Indeed, it was these mature atherosclerotic lesions, where we observed the most significant differences.

The observed phenotype represents a so-called stable atherosclerotic lesion since unstable lesions are characterized by low numbers of SMCs and a reduced fibrous cap covering the lesion. Moreover, these lesions contain increased numbers of inflammatory cells [39]. The enhancement of extracellular matrix deposition and decrease of macrophage involvement found in the lesions of TGF-ß1 overexpressing mice is in full accord with the pro-fibrotic properties of TGF-ß also seen in other experimental set-ups [6], [40], [41] and the strong anti-inflammatory function of the cytokine as demonstrated by the phenotype of TGF-ß knock-out mice [42].

Of course, the genetic manipulations of mice used for atherosclerosis research might influence the effects of the candidate gene of interest. However, as atheroprotective effects of TGFß also could be observed in LDLR^−/−^ mice [9], [43], it is unlikely that impairment of reverse cholesterol transport is the only context in which our observations are seen.

Are macrophages themselves the main target accounting for the observed phenotype? We are currently investigating whether ablation of TGFßR2 signalling in macrophages might influence atherogenesis. For macrophage-specific TGFß1 ablation a dominant negative (dn) operating truncated type II TGFß receptor cDNA (dn TGFßR2) [44] was placed under the transcriptional regulation of the SRA promoter/enhancer element. The resulting SRA-dnTGFßR2 fusion gene shows macrophage-specific overexpression of a dominant negative operating truncated type II TGF ß receptor which lacks the cytoplasmic serine/threonine kinase domain and thus the ability of TGFß1 signal transduction. Although macrophages principally have been shown to express TGFß1 receptors [45] and TGFß is considered as a potent macrophage-modulating agent [46], our preliminary results obtained in this animal model failed to detect any effect of transgenic macrophage-specific overexpression of a dominant negative acting TGFßR2 molecule on both lesion area and composition of murine atherosclerotic lesions. Hence, at least under conditions of physiological TGFß expression, lesion macrophages are obviously relatively insensitive to TGFß type II receptor dependent signalling. However, this result cannot necessarily be transferred to the SRA-TGFß1 overexpression model used in the current manuscript. Currently we investigate whether the lack of any pro- or anti-atherosclerotic effects in SRA-dnTRFßR2 mice with macrophage-specific ablation of TGFß signalling is mediated by a reduction of specific receptor expression. This regulatory mechanism has already been observed during the transition of monocytes to activated macrophages as mediated by other inflammatory stimuli [47], and is also known to restrict TGFß responsiveness of human smooth muscle cells isolated from atherosclerotic lesions [48]. In any case, the effects of macrophage-specific TGFß1 overexpression as described in the current study must not necessarily be induced by TGFß dependent signalling of macrophages, but could also be mediated by other cell types involved in atherogenesis, of which SMCs are the primary candidates.

Forkhead box protein P1 (FoxP1), a downstream target of TGF-ß signalling, might play an important role. As for the increase of SMC infiltration and collagen deposition, FoxP1 has been shown to be expressed by different cell types in atherosclerotic lesions and to be associated with more stable plaque characteristics and intraplaque TGF-ß signalling. Notably, *in vitro* stimulation of SMCs with TGF-ß resulted in increased FoxP1 levels [49]. With regard to the decrease of macrophage content, FoxP1 has been shown to impair the transition of monocytes towards macrophages and acts as a repressor of c-fms, which codes for the receptor of the differentiation stimulating growth factor M-CSF (Macrophage Colony Stimulating Factor) [50]. In short, there may be a negative feedback loop of macrophage-derived TGF-ß: since *in vitro* stimulation of SMCs with TGF-ß results in increased FoxP1 levels, FoxP1 in turn might decrease monocyte recruitment and differentiation. The decrease of macrophages itself leads to more stable atherosclerotic lesions. In future studies, it may be interesting to determine whether increased numbers of SMCs are due to proliferation of *in situ* native cells or increased recruitment of bone marrow-derived SMCs. If the latter is true, it would be interesting to note whether alterations in TGF-ß-receptor or TGF-ß1 expression in these cells could modulate these observed findings.

Summarizing, the experimental data of the current study demonstrates an antiatherogenic effect of macrophage-derived TGF-ß1 in atherosclerosis. Further studies will have to specify the cellular mechanisms underlying the effects of the overexpression of active TGF-ß1 in murine macrophages. Our current results clearly point to a role of the cytokine in atherogenesis and provide a likely avenue for future research that may lead to novel pharmacological therapies for the treatment or prevention of atherosclerosis.
